# Fermented Vegetables as a Source of Psychobiotics: A Review of the Evidence for Mental Health Benefits

**DOI:** 10.1007/s12602-025-10592-5

**Published:** 2025-05-22

**Authors:** Eman Shawky, Shelini Surendran, Rasha M. Abu El-Khair

**Affiliations:** 1 Department of Pharmacognosy, Faculty of Pharmacy, Alexandria University, Alkhartoom Square, Alexandria, 21521 Egypt; 2 Faculty of Health and Medical Sciences, University of Surrey, Guildford, UK; 3 Pharmacognosy Department, College of Pharmacy, Arab Academy for Science, Technology and Maritime Transport, Alexandria, Egypt

**Keywords:** Fermented vegetables, Gut-brain axis, Microbiome, Mental health, Psychobiotics

## Abstract

The human gut microbiome, comprised of trillions of microorganisms, plays a pivotal role in both physical and mental health. Recent research underscores the intriguing connection between gut bacteria and mental well-being, leading to the emergence of psychobiotics—microbes with mental health benefits. This review aims to explore fermented vegetables, a traditional dietary staple experiencing renewed interest, as a potential source of psychobiotics. Fermentation alters the microbial composition of vegetables, enriching them with beneficial bacteria such as *Lactobacillus* and *Bifidobacterium*. Various fermented vegetables, including kimchi, sauerkraut, and tempeh, host distinct bacterial communities. The review investigates how these psychobiotics may impact mental health through the gut-brain axis, a communication network between the gut and the central nervous system. Possible mechanisms encompass neurotransmitter modulation (e.g., serotonin, GABA), inflammation reduction and immunity modulation, and stress response enhancement through the hypothalamic-pituitary adrenal (HPA) axis. Clinical studies exploring the influence of fermented vegetables on mental health outcomes, including anxiety, depression, and cognitive function, are critically evaluated. The review assesses the efficacy of different fermented vegetables and probiotic strains while recognizing limitations in existing research and the necessity for further investigation.

## Introduction

Mental health disorders, including anxiety and depression, are among the leading causes of disability worldwide, affecting millions of individuals and significantly diminishing quality of life. These conditions impose an increasing burden on healthcare systems, necessitating the exploration of novel, accessible, and safe therapeutic approaches. Traditionally, psychiatric disorders have been managed with pharmacological and psychological interventions; however, emerging research suggests that gut microbiota may play a crucial role in mental health regulation through the gut-brain axis, a bidirectional communication system linking the gastrointestinal system with the central nervous system. This connection has led to the development of the concept of psychobiotics—microorganisms that, when ingested in adequate amounts, exert beneficial effects on mental health [1]. The term psychobiotics was first introduced by Dinan et al. and initially referred to live microorganisms with mental health benefits. Over time, the definition has expanded to include not only probiotics but also prebiotics and fermented foods that can positively influence brain function. Although much of the current research on psychobiotics has focused on dairy-based probiotics, such as yogurt and kefir, fermented vegetables have garnered increasing attention for their unique probiotic composition and potential mental health benefits [2]. Unlike dairy-based probiotic sources, fermented vegetables provide a diverse and naturally occurring array of beneficial bacterial strains, many of which are involved in crucial metabolic and neurochemical processes.

Fermentation is a natural metabolic process that enhances the microbial composition of vegetables, enriching them with beneficial bacteria such as Lactobacillus plantarum, Leuconostoc mesenteroides, and Bifidobacterium species. These probiotic strains contribute to mental health through multiple mechanisms, including the production of neurotransmitters (e.g., serotonin and gamma-aminobutyric acid [GABA]), modulation of inflammation, and regulation of the hypothalamic–pituitary–adrenal (HPA) axis [3].

The gut-brain axis has been extensively studied as a key mechanism underlying psychobiotic activity. Research suggests that psychobiotics influence mood and cognitive function through three primary pathways: (1) modulation of neurotransmitters such as serotonin and dopamine, (2) reduction of systemic inflammation via suppression of pro-inflammatory cytokine levels, and (3) regulation of the stress response through modulation of the HPA axis. Notably, fermented vegetables such as kimchi, sauerkraut, and tempeh contain high concentrations of lactic acid bacteria, which may confer psychobiotic properties. The bioactive compounds produced during fermentation, such as short-chain fatty acids (SCFAs) and polyphenols, may further enhance their potential benefits for mental health [4].

Despite these promising attributes, the role of fermented vegetables as psychobiotics remains underexplored compared with probiotic supplements and dairy-based fermented foods. Limited clinical evidence exists regarding their efficacy in managing mental health disorders, highlighting the need for a comprehensive evaluation of their potential benefits. Clinical studies on psychobiotics have shown mixed results, with some demonstrating improvements in depressive and anxiety symptoms, whereas others indicate a need for strain-specific research to optimize effectiveness. Moreover, standardization in probiotic strain identification, fermentation processes, and dosing remains a challenge in translating psychobiotic interventions into clinical practice [5, 6].

This review aims to bridge this gap by critically examining the existing evidence on fermented vegetables as a source of psychobiotics. It explores their microbial composition, mechanisms of action via the gut-brain axis, and their potential impact on psychiatric conditions such as anxiety and depression. Additionally, the review identifies research gaps and future directions to establish the clinical relevance of fermented vegetables in mental health management.

## Comparison of Fermented Vegetables and Dairy-Based Probiotics as Psychobiotics

Traditional probiotic sources, such as dairy-based products (e.g., yogurt, kefir, and fermented milk), have been extensively studied for their psychobiotic effects. These products are rich in lactic acid bacteria (LAB), such as *Lactobacillus* and *Bifidobacterium* species, which have demonstrated benefits in regulating the gut-brain axis, modulating neurotransmitters like serotonin and GABA, and reducing inflammation [7].

Although dairy-based probiotics are well-documented, fermented vegetables offer a unique and complementary psychobiotic profile. Unlike dairy-based products, fermented vegetables contain plant-derived bioactive compounds, such as polyphenols and dietary fiber, which further enhance gut microbiota diversity and influence mental health through anti-inflammatory and antioxidant mechanisms [8]. Additionally, fermented vegetables provide a broader range of naturally occurring LAB strains without the presence of lactose or dairy allergens, making them suitable for individuals with lactose intolerance or dairy allergies [9].

Moreover, the fermentation process in vegetables yields different metabolic byproducts, including short-chain fatty acids (SCFAs) and bioactive peptides, which may exert additional psychotropic effects by modulating the gut microbiome [10]. Compared with dairy-based probiotics, which primarily rely on added bacterial cultures, fermented vegetables undergo a natural fermentation process, allowing for the growth of diverse beneficial microbial communities that can adapt to environmental and dietary conditions.

Thus, although both dairy-based probiotics and fermented vegetables contribute to mental health benefits through psychobiotic mechanisms, the latter offers an alternative, plant-based source that may provide broader gut microbiota modulation and additional health benefits beyond conventional probiotics. Further comparative research is necessary to determine the specific advantages of each in clinical applications for mental health.

## Overview of Fermented Vegetables as Psychobiotics

The traditional approach to vegetables as a source of vitamins, minerals, and, more recently, as a source of fiber for human health has overlooked the fact that vegetable material from the whole plant is a major resource for fermented food products, as well as substrates and habitats for postharvest spoilages by a wide range of microorganisms [9].

Fermentation as a process has existed for many centuries. It was first described as an important process for wine and beer making. Initially, it flavors and preserves food, normally through the growth of desirable mild organisms and/or the inhibition of the growth of others (Steinkraus, 1997). Fermentation occurs in all vegetables; however, the product of the fermented vegetables changes depending on the vegetables’ chemical composition and the depth to which the sugar is catabolized. Fermentation is an age-old practice that has been used for many vegetables before they were pickled. Fermentation was a practice used to extend the life of vegetables, such as the fermentation of Kimchi from Korea [11].

Fermented foods are major food items in several areas of the world. Fermented vegetables are excellent carriers for lactic acid bacteria (LAB) fermentation. Raw vegetables serve as natural substrates for LAB fermentation. During this process, LAB metabolize the naturally occurring sugars in the vegetables, producing lactic acid, which preserves the food and enhances its probiotic content. The composition and growth of LAB depend on the vegetable type, fermentation conditions, and the presence of naturally occurring or added microbial strains. Once fermentation is complete, the vegetables become enriched with LAB, making them an excellent source of probiotics with potential psychobiotic properties.

The preservation of vegetables through LAB fermentation is an age-old technology that is still practiced worldwide. The majority of the fermented products are still manufactured at home and are simply a means of preserving the harvest [8]. The oldest and the most widespread method of food fermentation is the spontaneous method, in which microorganisms already present in the raw materials or the air are allowed to grow on and in the food. This method of fermentation is still widely used in the production of tempe, traditional fermented sausages, some types of cheese, and yogurt [12]. Another method of fermentation is by using a natural starter culture. It is almost the same as the spontaneous method, but the number and type of the microorganisms involved in the fermentation can be more controlled. It is done by the addition of a small portion of previously successfully fermented food (back sloping) or in certain cases by the addition of parts of the crop with a purpose to encourage fermentation by beneficial microorganisms before spoilage microorganisms or pathogens attack it [13]. The most modern method of fermentation is by using a pure culture starter. This method is done by the addition of selected and pure cultures of microorganisms in high concentration in the food, which will be fermented [14].

The most famous fermented vegetables are sauerkraut (Europe), kimchi (Korea), and tsukemono (Japan). In the fermentation of vegetables, the main microbiological activity is the lactic acid bacteria fermentation [15]. These microorganisms convert sugar in the vegetables into various organic acids, mainly lactic acid. In the traditional fermentation of vegetables, these lactic acid bacteria come from the vegetables themselves or the materials used in the process. This is why sometimes the fermentation of the same vegetables using the same method but at a different time and place can result in a different taste. The lactic acid bacteria also play a part in keeping the vegetables from being spoiled by pathogens or other undesired microorganisms. This is, of course, different from the fermentation of beverages because there are some types of lactic acid bacteria that are pathogenic and can cause food poisoning [16].

The trend in present-day society has shifted from work that aims to conserve food through fermentation to a better understanding of the health benefits associated with consuming fermented foods. The importance of the microbiota of vegetables is increasingly being recognized. Although considerable attention has been given to the role of the microbiomes of the many edible fruits of the plant kingdom, ranging from bananas to oranges, and the diverse microbiomes associated with edible plant tissues such as nectars and saps, the attention given to the vegetable kingdom has been biased towards a focus on disease [12]. This is because of the overwhelming economic impact of diseases of vegetable crops, whether in the field or postharvest. In this context, the emphasis has been on understanding the role of causative agents in plant pathogens and how they may be controlled in an environmentally friendly manner using modern microbiological knowledge, rather than seeking to understand the complex and beneficial microbial assemblies associated with healthy vegetables [12].

## Types and Bacterial Communities of Fermented Vegetables

### Types of Fermented Vegetables

There are more than 300 different types of fermented vegetables (Table 1). Fermented vegetables are produced by lactic acid fermentation, in which the primary food material of the vegetable is converted to cell acids. The acid environment then prevents the growth of spoilage organisms and may inhibit the growth of pathogenic microorganisms [17]. The end product is a preserved food that has its specific type of pickled taste, which is determined by the microorganisms present and the food substrate being fermented. Both spoilage and lactic acid bacteria are involved in vegetable fermentations, with the latter generally being the more desirable fermenters. It must be noted here that not all fermented vegetables are prepared using salt and lactic acid fermentation [18]. Traditionally, fermented vegetables are considered to be a poor man’s food because the fermentations were considered to be simple and did not require the sophistication of other food fermentations. However, today there is a growing interest in traditional and ethnic foods and many of these have a vegetable ferment with a rich history and tradition [19].

**Table 1:** Fermented vegetables and their key probiotic strains

Fermented vegetable	Key probiotic strains	Region/origin
Sauerkraut	*Lactobacillus plantarum, Leuconostoc mesenteroides, Pediococcus pentosaceus*	Central Europe
Kimchi	*Lactobacillus plantarum, Lactobacillus kimchii, Leuconostoc mesenteroides*	Korea
Tempeh	*Lactobacillus plantarum, Pediococcus acidilactici, Bacillus subtilis*	Indonesia
Miso	*Aspergillus oryzae* (mold)*, Lactobacillus delbrueckii*	Japan
Kimchi (Radish)	*Lactobacillus plantarum, Leuconostoc mesenteroides, Weissella cibaria*	Korea
Kombucha	*Saccharomyces cerevisiae* (yeast)*, Lactobacillus casei, Bifidobacterium*	East Asia
Kvass	*Lactobacillus brevis, Lactobacillus sanfranciscensis*	Eastern Europe
Achar	*Lactobacillus plantarum, Leuconostoc mesenteroides* (varies depending on vegetables used)	India
Pickles (cucumber)	*Lactobacillus spp., Weissella spp.*	Various (globally)
Kimchi (Water Kimchi—Kimchi without napa cabbage)	*Lactobacillus brevis, Lactobacillus plantarum, Leuconostoc mesenteroides*	Korea
Sauerkraut (Beet)	*Lactobacillus plantarum, Leuconostoc mesenteroides*	Variation of Central European sauerkraut
Fermented bamboo shoot	*Lactobacillus plantarum, Pediococcus pentosaceus*	India

Kimchi is one of the most well-known traditional fermented foods still consumed in South Korea. Kimchi is fermented from a mixture of vegetables, which can include Napa cabbage, cucumber, radish, and scallion. It is then seasoned with a range of different spices. The final product is a well-balanced spicy/sour food. Kimchi is usually consumed during the winter months when freshly available vegetables are scarce [20]. Traditionally, the kimchi fermentation and storage process was performed in large earthenware pots partially buried in the ground. Although it is still common for households to have large kimchi pots, it is now more common to use a standard household refrigerator for fermentation [20]. Kimchi contains over 400 types of lactic acid bacteria, including *Weissella cibaria*, *Lactobacillus plantarum*, *Leuconostoc citreum*, and *Lactobacillus brevis*, and some types of kimchi were found to have high levels of lactic acid bacteria and bifidobacteria [21]. Lactic acid bacteria isolated from kimchi were tested for their bile salt hydrolase activity, and Lb. Brevis was found to have the highest bile salt hydrolase activity and is a potential probiotic strain [22]. Kimchi also contains beneficial LAB such as *Leuconostoc mesenteroides*, which has a high GABA (a naturally occurring amino acid, well known for its calming effect on the nervous system) producing ability [23].

Sauerkraut is made from shredded cabbage and salt, which are mixed together, allowing the natural juices of the cabbage to be released. Traditionally, this involves allowing the cabbage to sit for several days in an airtight container before being transferred to a second container. Storage for up to a month at room temperature allows the live bacteria population to further acidify the cabbage. Today, most store-bought sauerkraut is pasteurized and therefore lacks the live bacteria cultures known to have health benefits [24]. The end product is determined largely by the fermentation temperature. At temperatures below 15 °C, slow fermentation will occur, which produces a more acidic, longer-lasting product. At temperatures 18 °C and above, the product has a shorter shelf life but is less acidic. In both cases, the primary organism is *Leuconostoc mesenteroides*, which is a psychrotroph requiring a low temperature to grow. *Pediococcus* and *Lactobacillus* bacteria are also involved in the further breakdown of the sugars to lactic acid. If not hygienically produced, fungi and even *E. coli* have been found to grow on sauerkraut during fermentation and storage. This can be dangerous and in some cases deadly. However, the organic acids produced by the lactic acid bacteria produce a product of very low pH, creating a high bacteriological safety even at room temperature. This process can take up to 6 weeks, but sauerkraut can last many months in an airtight container at room temperature [25].

Fermented bamboo shoot usually refers to the pickled or fermented tender shoots of the bamboo (*Bambusa vulgaris*) plant and is commonly consumed in parts of Asia. Although it is often classified as a vegetable, bamboo is actually a type of grass, and the edible bamboo shoot is a highly nutritious, edible culm part of the bamboo plant. Lactic acid fermentation of bamboo shoot occurs spontaneously in brine and is traditionally carried out at ambient temperatures, for time periods ranging from a few days to several months [26]. In West Bengal, India, it is commercially prepared as a fermented product and is a popular delicacy in the region. The indigenous people residing in the bamboo-growing regions of the Darjeeling hills in West Bengal and Sikkim, India, have long embraced Mesu, a traditional fermented bamboo shoot product, which is typically free of added salt. Lactic acid bacteria isolated from fermented bamboo shoots in North-East India were identified as *Lactobacillus plantarum* and *Pediococcus pentosaceus*, both of which are heterofermentative LAB frequently associated with the fermentation of vegetables [27]. Although specific details on its fermentation microbiology and bacterial community are scarce, fermented bamboo shoots are a lesser-known, unique fermented food worthy of investigation for its potential health benefits.

#### Key Probiotic Strains in Fermented Vegetables

Compared with other food products such as dairy, certain fruits, and juices, there is a noticeable gap in research on specific probiotic strains present in fermented vegetables. A comprehensive review of the scientific literature indicates that fermented vegetables offer a broader spectrum of probiotics than some other food sources [28]. Common strains found in fermented vegetables include various species of *Lactobacillus plantarum*, an alpha-hemolytic *Streptococcus*, *Leuconostoc mesenteroides*, and *Pediococcus* species. *L. plantarum* is particularly prevalent as it has been isolated from a wide range of fermented food products and human saliva [28]. Lactobacillus plantarum is widely recognized for its health benefits and is a commonly found probiotic in fermented foods. Its beneficial effects include binding and removing toxins from the body, enhancing the immune system by increasing antioxidative agents, and preventing DNA and RNA damage caused by mutagenic compounds. Consequently, *L. plantarum* is a highly desirable probiotic, and fermented vegetables are a reliable source of this strain. This information is crucial for highlighting the significance of specific probiotic strains in fermented vegetables [29]. The various species of *Pediococcus* are also significant due to their ability to ferment different sugars, leading to the production of desirable end products, such as diacetyl from glucose. This diverse array of probiotics and their unique fermentation pathways contribute to the distinct flavors and nutritional benefits of fermented vegetables [30].

*Lactobacillus* is a key probiotic strain, primarily because it ferments the sugars in vegetables to produce lactic acid. This process is driven by a set of genes known as the lac operon, which encodes enzymes that metabolize the diverse sugars present in vegetables, converting them into lactic acid. Subsequently, these enzymes utilize the lactic acid produced to generate energy for the bacterium [31]. This leads to one of the primary differences between raw and fermented vegetables: an increase in acidity creates a noxious environment for spoilage organisms and pathogens, which makes preserving the vegetables much less difficult and makes it far less likely that the vegetables will spoil in the end result [32]. The various *Lactobacillus* species share the common ability to produce lactic acid as the primary or sole end product of their fermentative metabolism. Lactic acid is responsible for the distinctive sour taste of fermented vegetables and serves as a preservative by inhibiting the growth of harmful bacteria. Additionally, Lactobacilli can produce antimicrobial compounds such as bacteriocins, hydrogen peroxide, and acetic acid, which further suppress the growth of pathogenic bacteria. This enhances the safety and effectiveness of food preservation through fermentation [31]. *Lactobacillus plantarum* is one of the most prevalent species found in fermented foods like kimchi, sauerkraut, and pickles. It thrives in the low pH and high salt conditions typical of vegetable fermentations. This species is known for its strong probiotic and antioxidant properties and includes at least 10 strains that can adhere to the intestines. *L. plantarum* is also capable of surviving the digestive process, effectively delivering its probiotic benefits to the consumer. Fermentation time significantly impacts the probiotic potential of *Lactobacillus*; studies have shown that longer fermentation periods increase lactic acid and vitamin C content, thereby enhancing the overall probiotic effectiveness of the fermented product [33].

*Bifidobacterium* comprises 32 species, but only a few are commonly found in fermented vegetables. Individuals following a specific carbohydrate diet have reported that the beneficial bacteria in fermented vegetables can alleviate symptoms of Crohn’s disease, especially when other treatments have failed. This effect is likely partly due to the production of short-chain fatty acids and the absorption of ammonia, processes in which *B. infantis* may play a role. However, the high production of acetate and lactate can influence the acidity of fermented vegetables, which, at excessively high levels, might have negative effects [34]. Due to the general lack of information regarding which *Bifidobacterium* species is present in fermented vegetables, this is an area that largely requires more research.

## Mechanisms of Action: How Psychobiotics from Fermented Vegetables May Influence Mental Health

The gut-brain axis is the biochemical signaling pathway that connects the central nervous system with the peripheral digestive system. This bidirectional system is regulated by the CNS and has an effect on brain function and behavior [35]. The gut-brain axis is a developing field in neuroscience that has highlighted the potential for gut microbes to influence brain function. The gut-brain axis works via humoral, immune, and neural communication between the gut and the brain. Research has suggested that the brain can in fact influence the progress of a disease in the gut [36]. Preclinical studies on affective psychobiotics have demonstrated their anxiolytic and antidepressant-like effects in three main ways: by directly influencing neurotransmission, through the regulation of the hypothalamic pituitary adrenal axis, and by countering the effects of stress and inflammation [37]. In this section, we will discuss how probiotics can mediate through the gut-brain axis to increase brain health and ultimately suggest a mechanism by which fermented food, such as pickles, may have a direct effect on mental health.

### Influence on Neurotransmitter Production

It has long been thought that much of the potential of fermented foods to benefit mental health comes from the production of various non-digestible (to humans) compounds such as essential fatty acids and phytoestrogens, but recent investigation suggests that the capacity for some fermented foods to ameliorate symptoms of depression may also be due to the production of lactic acid bacteria, which in a similar fashion to the microbiota in the gut, show an ability to manipulate brain chemistry and influence mood [38]. Changes in brain chemistry can not only be initiated through the vagus nerve from the gut itself but can also take place through the direct synthesis of neurotransmitters within the CNS.

Evidence supports the idea that microorganisms have the capability to generate neuroactive chemicals, thereby facilitating direct communication between the gastrointestinal tract and the central nervous system (CNS). For instance, *Lactobacillus* spp. and *Bifidobacterium* spp. are capable of synthesizing neurotransmitters like acetylcholine and gamma-aminobutyric acid (GABA), whereas *Streptococcus* spp., *Enterococcus* spp., and *Escherichia* spp. produce serotonin, dopamine, and norepinephrine, respectively. [39].

Serotonin is a neurotransmitter that is synthesized from the amino acid tryptophan. It has been implicated in a vast array of physiological and cognitive processes, including mood, emotional regulation, sleep, learning, and memory [40]. The transportation of tryptophan over the blood–brain barrier is a competitive mechanism among other amino acids in the blood that rely on the same transporter. This means that manipulation of diet to increase the levels of tryptophan in the blood can result in increased levels of tryptophan being transported in the brain and therefore increased serotonin production. Once in the brain, tryptophan is converted to 5-hydroxytryptophan by tryptophan hydroxylase, an enzyme that is the rate-limiting step in serotonin production. This is then converted to serotonin by the enzyme aromatic l-amino acid decarboxylase [40].

Gut microbes possess the capability to metabolize the essential amino acid tryptophan, using it as a precursor for the synthesis of various compounds such as indole, serotonin, and melatonin. This process effectively limits the availability of tryptophan for the host. Specifically, certain strains like *Pseudomonas* spp. are capable of synthesizing serotonin from tryptophan, serving roles in toxicity and intercellular signaling. Consequently, the reduction in circulating tryptophan due to gut microbiota activity impacts serotonergic transmission and the function of both the central nervous system (CNS) and enteric nervous system [7]. Microbiota-induced tryptophan metabolism also influences the production of tryptamine through the action of tryptophan decarboxylase, which in turn modulates cellular responses to serotonin by enhancing its secretion from enterochromaffin cells. It is widely recognized that over 90% of serotonin resides in the enterochromaffin cells situated in the gastrointestinal tract, where it is triggered to release in response to signals from the gut lumen, thereby regulating gut movement and sensation.

Given that significant quantities of lactic acid bacteria and *Bifidobacteria* are found in the intestines, it is theoretically possible that they may be able to influence mood and behavior by enhancing serotonin production in the intestines as a result of increased tryptophan availability. This, in turn, could result in increased serotonin production in the brain [41]. Metabolites or components derived from the gut microbiota prompt the synthesis and release of serotonin, and heightened serotonin production in the gastrointestinal tract could lead to decreased levels of tryptophan in the bloodstream, consequently reducing serotonin synthesis in the brain [42]. Therefore, the catabolism of tryptophan associated with gut microbiota emerges as a critical regulator of the brain-gut axis, underscoring the profound influence of microbial activity on neurological function and behavior.

Studies in preclinical models have illustrated the capacity of fermented soy-based foods like tempeh to influence the expression of serotonergic genes. For instance, zebrafish fed with tempeh exhibited increased expression of genes involved in serotonin transportation, synthesis, and signaling in the brain, namely tph1b, tph2, and slc6a4a genes [43]. Gamma-aminobutyric acid (GABA) stands out as a crucial inhibitory neurotransmitter within the central nervous system (CNS), with its receptors widely distributed throughout. GABAergic neurotransmission plays a pivotal role in diverse CNS functions, including behavior, pain perception, and regulation of sleep. Moreover, it extends its influence to gastrointestinal tract functions, such as modulating intestinal motility, gastric emptying, nociception, and acid secretion [44].

Microbes within the gut environment can either consume or produce GABA. The gut microbiota exerts an influence over circulating GABA levels, with notable reductions observed in luminal serum levels among germ-free (GF) animals. This underscores the intricate relationship between gut microbes and the regulation of GABA levels, highlighting the potential impact on neurological and gastrointestinal functions. Regarding consumption, the GABA shunt pathway, which converts GABA to succinate through the tricarboxylic acid (TCA) cycle, holds significance. For instance, *Escherichia coli* is capable of producing GABA from specific carbon and nitrogen sources, although the precise function of GABA-consuming microbiota remains elusive. In contrast, the systemic role of GABA has been extensively documented, with various microbial strains known for their ability to synthesize GABA [45].

Members of the *Bifidobacterium* and *Lactobacillus* genera play integral roles in gamma-aminobutyric acid (GABA) synthesis. Notably, *Lactobacillus rhamnosus* (JB-1), one of the most frequently studied strains, has shown promising results in mitigating depressive symptoms and anxiety when administered to mice. These effects were linked to vagus nerve activity modulation and observed alterations in cerebral GABAergic activity [46]. In an investigation, oral administration of *Bifidobacterium* breve NCIMB8807 pESHgadB in a rat model demonstrated reduced GABA synthesis and heightened tolerance to visceral pain. This outcome was attributed to the upregulation of glutamate decarboxylase B expression [47].

Although there are limited preliminary studies in humans on this topic, it is anticipated that modulating the human microbiota could influence GABA levels. Dietary adjustments can significantly impact the function and composition of the microbiota. For instance, the ketogenic diet has been associated with heightened GABA levels in cerebrospinal fluid and notable symptom alleviation in children with treatment-resistant epilepsy [48].

### Reducing Inflammation and Modulating the Immune System

Dysbiosis refers to an imbalance in both the composition and function of the native microbial community within the gastrointestinal tract. Dysbiosis possesses the capacity to trigger an inflammatory response that may extend into the bloodstream and subsequently affect the brain [49]. The significance of inflammation in various chronic illnesses, such as depression, should not be underestimated, as specific data underscore its crucial role. Apart from cytokines, other mediators can relay signals from the gastrointestinal tract to the brain via the vagus nerve [50]. Various altered conditions can contribute to the emergence of neuropsychiatric symptoms induced by gut dysbiosis compared with control individuals. These circumstances encompass alterations in microbial composition and metabolite production, subsequently influencing the levels and synthesis of neurotransmitters such as serotonin, dopamine, noradrenaline, and glutamate. Consequently, dysbiosis disrupts the balance of the gut-brain axis, leading to adverse effects on brain mechanisms [10]. Dysfunction in the gastrointestinal system triggers sustained systemic inflammation, compromising the structural integrity of the blood–brain barrier by inducing degradation of tight junction and anchor proteins in specific regions like the frontal cortex, hippocampus, and striatum, consequently affecting brain function. Consequently, this disruption in brain function occurs [51]. The increased permeability of the blood–brain barrier facilitates the movement of immune cells and harmful microbial metabolites into the brain, intensifying the release of cytokines, chemokines, and endocrine messengers associated with stress within the brain parenchyma. Indeed, the imbalance in gut bacteria disrupts the signaling between the gut and the brain, initiating the pro-inflammatory process. This activation is directly linked to symptoms associated with neuropsychiatric disorders (NPS) [51].

Two possible mechanisms by which psychobiotics may influence mental function are through the reduction of pro-inflammatory cytokines and oxidative stress. It has been proposed that increased levels of certain beneficial bacteria may downregulate the production of pro-inflammatory cytokines through the production of anti-inflammatory cytokines. It has been suggested that psychotropic medications may dampen the stress-induced production of pro-inflammatory cytokines, which is often enhanced in major depression and has been suggested to contribute to the symptomatology of the illness [52]. Pro-inflammatory cytokines have been found to induce indoleamine 2, 3-dioxygenase (IDO), an enzyme that catabolizes tryptophan (the amino acid precursor to serotonin) into kynurenine and subsequently neurotoxic quinolinic acid. Inflammation and the kynurenine pathway have been linked with depression and anxiety, and it is hypothesized that a tryptophan-depletion–induced decrease in serotonin may be a contributory factor [53]. An increase in the prevalence of lactobacilli and other tryptophan-consuming bacteria may also indirectly affect tryptophan availability through competition with the host for the substrate [54].

Psychobiotics may also reduce inflammation via the production of SCFAs in the gut. SCFAs can exert profound anti-inflammatory effects on both the immune and non-immune cells. They do this by inhibiting the synthesis of pro-inflammatory cytokines. Data have suggested that inflammation is associated with a subset of depression and anxiety symptoms. Inflammation also reduces the availability of tryptophan, the amino acid precursor to serotonin, through an increased activation of indoleamine 2,3-dioxygenase [54]. This provides a feasible link between inflammation and decreased serotonin function (another identified cause of depression). Tryptophan is metabolized into kynurenine by the enzyme indoleamine 2,3-dioxygenase (IDO). Reduced availability of tryptophan leads to decreased serotonin production and increased kynurenine production, which is linked to the development of depression. Because tryptophan is also metabolized along the kynurenine pathway in the liver, increasing tryptophan availability by inhibiting IDO can potentially be supported through dietary tryptophan, particularly when there are issues with tryptophan availability in the brain [55]. Therefore, reducing inflammation could be a very effective method of prevention and treatment of depression and anxiety. The anti-inflammatory action of SCFAs and the subsequent impact on tryptophan metabolism provides a possible target for psychobiotics in affective mental health and disorders of mood [52].

The immune system is a complex network of cells, tissues, and organs that work in unison to protect the body from infection. The body’s immune response can either be innate or adaptive, with the adaptive response suggesting recognition of the pathogen and a learned reaction [43]. T-regulatory cells (T-regs) are a vital component of the immune system in maintaining peripheral tolerance and preventing autoimmunity. They do this by suppressing the immune response to self-antigens or allergens to stem an immune-mediated inflammatory response. This can be beneficial to mental health through normalizing the immune response, which may be hypothesized to be the case in certain mental health disorders. A normal immune response can activate behavioral and cognitive changes to recuperate health and fight infection; however, continuation of such symptoms over a long period of time can manifest as depression or anxiety [56]. This is demonstrated in sickness behavior, a state of altered activity, arousal, appetite, and ability to focus, closely resembling symptoms of depression and anxiety. Sickness behavior occurs as a direct effect of cytokines on the brain and is a key mechanism of action by which inflammation can result in mental disorders. T-reg activity in preventing immune-mediated inflammatory response can also be beneficial in preserving mental health by acting on the blood–brain barrier to preserve cognitive function and mental status [57].

Microbial metabolites possess the capacity to modulate the immune system, whether through systemic circulation or via the vagus nerve. Recent studies have shed light on this phenomenon, highlighting the role of microbial products in immune regulation [58]. Moreover, emerging evidence underscores the presence of receptors for bacterial cell wall components and immunostimulants like peptidoglycan and lipopolysaccharides in the brain. This suggests a profound impact of the gut microbiota on host brain function and behavior [59].

Various components found in fermented foods are currently under investigation for their potential to modulate the immune system. Bacterial cellular components present in these foods have demonstrated the capacity to stimulate the release of interleukin-10 (IL-10) from dendritic cells and CD4 + T cells [43]. One potential mechanism through which fermented foods may exert immunomodulatory effects is by activating the hydrocarboxylic acid receptor (HCA3R) upon consuming LAB-fermented products. For instance, sauerkraut, which is also abundant in LAB, has been shown to induce a chemotactic response from monocytes by the presence of d-phenylacetic acid, a potent agonist of the HCA3R receptor [60]. Genome-wide association analyses on a large scale have indicated that LAB strains detected in the human gut are likely derived from dietary sources [61].

### Improving Stress Response Through the Hypothalamic-Pituitary Adrenal (HPA) Axis

Stress, particularly chronic unpredictable stress, is a key ethological model to induce depression and anxiety in rodents. Emerging research suggests a potential link between fermented foods and the hypothalamic–pituitary–adrenal (HPA) axis, a complex neuroendocrine system involved in the body’s response to stress. The HPA axis is composed of the hypothalamus, pituitary gland, and adrenal glands, which work in concert to regulate the body’s stress response through the release of hormones like cortisol [62].

Feeding murine models fermented foods such as red bean tempeh led to a reduction in anxiety-like behaviors and corticosterone levels under stress conditions [63]. Comparable reductions in depressive symptoms and anxiety-like behavior, along with decreased plasma corticosterone levels and inflammatory markers such as NF-Κb, TNF-α, and IL-6, were noted in the colons of mice given fermented ginseng extract supplementation for a duration of 5 days [43].

Studies suggest that gut microbes play a vital role in the HPA axis and the regulation of depression and anxiety-like behaviors. Probiotics and prebiotics may alter the production and plasticity of brain neurotransmitters [43]. Germ-free mice have been shown to have altered levels of 5-HT, DA, and NE and exhibit changes in associated behaviors. Mice given *B. longum* have been shown to have increased levels of brain-derived neurotrophic factor (BDNF) in the hippocampus [64]. BDNF has an established role in the pathogenesis of depression and anxiety and is a mediator for the effects of antidepressant treatments as well as being a key factor for the repair of HPA axis damage caused by chronic stress. BDNF expression is modulated by varying the activity of the cAMP response element (CREB). Both *B. longum* and FOS diet-treated mice had an increased expression of CREB in the hippocampus [65]. Many mechanisms behind how microorganisms affect the gut-brain axis and stress-related behaviors are still to be realized. Studies in the future may identify a number of bacterial-derived neuroactive compounds that may have effects at the BBB or directly affect the enteric and autonomic nervous systems. A comprehensive understanding of these mechanisms may lead to the use of specific microorganisms or even gene therapies to alleviate stress-related psychiatric disorders [66].

## Strain-Specific Contributions to Psychobiotic Effects

Specific bacterial strains present in fermented vegetables contribute to mental health through various mechanisms, including neurotransmitter modulation, immune regulation, and HPA axis stabilization. Different strains of Lactobacillus, Bifidobacterium, and Leuconostoc play key roles in influencing brain function and reducing stress-related symptoms. One of the primary ways psychobiotics exert their effects is through neurotransmitter modulation. Certain *Lactobacillus* species, such as *Lactobacillus plantarum* and *Lactobacillus brevis*, found in fermented vegetables like kimchi and sauerkraut, are capable of producing gamma-aminobutyric acid (GABA), an inhibitory neurotransmitter known for its calming and anxiolytic effects [47]. Similarly, *Bifidobacterium infantis* influences tryptophan metabolism, increasing serotonin availability in the gut and brain, which is linked to improved mood and emotional regulation [43]. Additionally, some *Enterococcus* and *Lactobacillus* species have been shown to synthesize dopamine, a neurotransmitter associated with motivation and reward processing [40]. These strain-specific activities suggest that the presence of these bacteria in fermented vegetables may help regulate mood, reduce anxiety, and enhance cognitive function. Beyond neurotransmitter production, immune system modulation is another key function of psychobiotics found in fermented vegetables. *Leuconostoc mesenteroides*, commonly found in sauerkraut and kimchi, produces short-chain fatty acids (SCFAs) such as butyrate, which has anti-inflammatory properties and enhances gut barrier function [46]. Butyrate plays a critical role in maintaining gut integrity and reducing systemic inflammation, which has been linked to mood disorders such as depression and anxiety. Additionally, *Lactobacillus plantarum* has been shown to suppress pro-inflammatory cytokines like IL-6 and TNF-α, while promoting anti-inflammatory cytokines such as IL-10, contributing to a more balanced immune response [10]. Certain *Pediococcus* species, also present in fermented vegetables, help strengthen gut barrier integrity by enhancing tight junction proteins and mucin production, reducing gut permeability, and preventing the translocation of inflammatory molecules into circulation [55]. These findings indicate that consuming fermented vegetables may help mitigate chronic inflammation, which is often associated with neuropsychiatric conditions. In addition to immune regulation, fermented vegetable-associated psychobiotics have been implicated in stress regulation via the hypothalamic–pituitary–adrenal (HPA) axis. *Lactobacillus rhamnosus (JB-1)*, which has been detected in naturally fermented vegetables, has been shown to reduce corticosterone levels and anxiety-like behaviors in animal models by modulating GABA receptor expression in the brain [63]. Additionally, *Bifidobacterium longum* supplementation has been found to reduce stress-induced cortisol secretion, suggesting that similar strains in fermented vegetables could exert stress-buffering effects [65]. Another important aspect of stress resilience is the role of brain-derived neurotrophic factor (BDNF), which supports neuroplasticity and brain function. Studies indicate that strains like *Lactobacillus plantarum* and *Bifidobacterium* species enhance BDNF levels, contributing to improved stress resilience and potential neuroprotection [66]. Taken together, these strain-specific contributions illustrate the diverse mechanisms through which fermented vegetables exert psychobiotic effects. Although dairy-based probiotics have been widely studied, fermented vegetables provide a naturally diverse bacterial profile enriched with unique strains that influence neurotransmitter balance, immune function, and stress resilience. Further research is needed to determine the optimal bacterial compositions and fermentation conditions that maximize these mental health benefits. By identifying and characterizing these bacterial strains, future psychobiotic interventions could be tailored to support mental health more effectively.

## Clinical Studies on Fermented Vegetables and Mental Health

### Impact of Fermented Vegetables on Anxiety

Anxiety disorders, characterized by excessive worry and fear, affect millions worldwide. Several studies have explored the association between fermented food consumption and anxiety symptoms. For instance, Hilimire et al. conducted a cross-sectional study involving 710 students, revealing an intriguing association between fermented food consumption and social anxiety, particularly in individuals with higher neuroticism. This suggests a potential role of fermented foods in modulating mood and anxiety [67] (Table 2).

**Table 2:** Clinical studies on the psychobiotic effects of fermented vegetables

Fermented food used and its dose	Participant number	Outcome measures	Key findings	Reference
Puffed fermented Korean ginseng extract-containing drink (10 mg of ginsenosides)	40 adult workers aged 30 to 60 years	Stress, fatigue, and sleep	Improvement in fatigue-inertia was observed in the group consuming puffed fermented Korean ginseng extract-containing drink	[68]
Psychobiotic diet (high in prebiotics and fermented foods)	45 adults	Microbial profile, mental health outcomes	Reductions in perceived stress observed in the group consuming psychobiotic diet, with stronger decreases in perceived stress observed with higher adherence to the diet	[69]
Diet rich in fermented food and high in dietary fiber	16,572 individuals	Depression, physical health indicators	Consumption of probiotic foods and higher intake of fiber associated with lower levels of depressive symptoms, mediated through improvements in physical health	[70]
Fermented food	372 medical students	Depressive and anxiety symptoms	Higher fermented food consumption is associated with more depressive and anxiety symptoms in psychiatrically healthy medical students under psychological stress. Psychiatrically ill individuals benefited more from fermented food consumption in terms of alleviating depressive symptoms	[71]
Fermented food and food-derived prebiotics	372 medical students	Cognitive performance, depressive, and anxiety symptoms	No relationship was observed between cognitive performance under stress and either fermented food or food-derived prebiotics consumption. High intake of fermented food is associated with more severe depressive and anxiety symptoms under stress	[72]
Tempeh A and Tempeh B (100 g/day)	90 respondents	Cognitive improvement	Both Tempeh A and Tempeh B consumption are associated with improved global cognitive function in older people with mild cognitive impairment. Tempeh A consumption is also associated with improvement in the language domain	[73]
Fermented food during pregnancy, especially miso	72,624 mother–child pairs (child aged 1 year)	Infant sleep duration	Maternal intake of fermented food during pregnancy is associated with a reduced risk of infant sleep duration of less than 11 h	[74]
Fermented foods (likely containing probiotics)	*N* = 710 (445 female)	Self-report measures of fermented food consumption, neuroticism, and social anxiety	Higher frequency of fermented food consumption was associated with fewer symptoms of social anxiety, particularly for individuals high in neuroticism. Suggested that fermented foods containing probiotics may have a protective effect against social anxiety symptoms, especially for those at higher genetic risk	[67]

Another study aimed to assess the efficacy of a puffed fermented Korean ginseng extract-containing drink in alleviating stress, fatigue, and sleep issues over an 8-week period among adult workers aged 30 to 60 years. The results, based on the efficacy analysis set of 38 subjects, did not reveal any significant differences between the groups in terms of efficacy. However, in a subgroup analysis of 11 middle-aged and older subjects aged 50 years or more, improvements in fatigue-inertia from baseline to week 4 were significant in the puffed fermented Korean ginseng extract-containing drink group compared to the placebo group (*P* < 0.05). The study confirmed that the puffed fermented Korean ginseng extract-containing drink can improve mental fatigue, especially in middle-aged and older subjects [68].

The study by Karbownik et al. examined the potential benefits of probiotic therapies and fermented food diets for improving mental health, focusing on a cohort of medical students facing psychological stress, including some with chronic psychiatric conditions. Fermented food consumption was assessed using 7-day dietary records, whereas depressive and anxiety symptoms were evaluated using standardized questionnaires. Among psychiatrically healthy medical students (*n* = 372), higher consumption of fermented foods was associated with increased depressive and anxiety symptoms. Conversely, among psychiatrically ill medical students, there was a significant negative correlation between fermented food intake and depressive symptoms. In summary, individuals with psychiatric conditions may benefit from consuming fermented foods to reduce depressive symptoms, whereas no such association was observed in healthy individuals [71].

Another study aimed to investigate the effects of a psychobiotic diet, rich in prebiotics and fermented vegetables, on both the microbial profile and mental health outcomes in a healthy human population in Ireland. The results indicated that the psychobiotic diet intervention led to reductions in perceived stress, with a 32% decrease observed in the diet group compared with a 17% decrease in the control group. Notably, higher adherence to the psychobiotic diet correlated with greater reductions in perceived stress. Although the microbial composition and function exhibited only subtle changes in response to the dietary intervention, significant alterations were observed in the levels of specific fecal lipids and urinary tryptophan metabolites. Furthermore, individuals on the psychobiotic diet who experienced greater microbial volatility demonstrated more pronounced changes in perceived stress scores [69]. These findings suggest that dietary interventions, particularly those targeting the microbiota, hold promise for reducing perceived stress in human populations. However, further research is needed to better understand the underlying mechanisms, including the role of the microbiota, in mediating the relationship between diet and mental health outcomes.

Another study aimed to investigate the potential association between maternal consumption of fermented foods during pregnancy and infant sleep duration at 1 year of age. Using data from The Japan Environment and Children’s Study (JECS), comprising 72,624 mother–child pairs, the researchers examined the relationship between maternal intake of fermented foods and infant sleep duration of less than 11 h.

The results of multivariable logistic regression analysis revealed that maternal consumption of fermented foods, particularly miso, during pregnancy was independently associated with a reduced risk of infant sleep duration of less than 11 h at 1 year of age [74].

### Impact of Fermented Vegetables on Depression

Depression, a leading cause of disability globally, has also been the focus of investigations into fermented food consumption. Certain strains of *Bifidobacterium* and *Lactobacillus* have displayed an ability to increase serotonin production in the CNS through increasing the availability of tryptophan, the amino acid precursor to serotonin, and through the direct production of serotonin by the lactic acid bacteria [75].

Multiple investigations have probed the link between fermented food consumption and depressive symptoms (Table 2). A study aimed to explore the relationship between a gut-health–promoting diet and depression, utilizing data from the National Health and Nutrition Examination Survey (NHANES) spanning from 2011 to 2018. The results revealed that consumption of probiotic fermented vegetables and a higher intake of dietary fiber were associated with lower levels of depressive symptoms. Furthermore, both subjective and objective physical health mediated the relationship between dietary factors and mild depressive symptoms. Specifically, subjective physical health acted as a mediator between high dietary fiber intake and reduced likelihood of severe depressive symptoms [70].

A comparative analysis involving 372 subjects between psychiatrically ill and healthy medical students provided intriguing insights into the differential effects of fermented food consumption on depressive symptoms. Psychiatrically ill individuals appeared to benefit more from fermented vegetables consumption, suggesting a potential avenue for targeted interventions [71].

### Impact of Fermented Vegetables on Cognitive Function

Cognitive health, particularly in aging populations, is a critical aspect of overall well-being. Several studies have investigated the impact of fermented foods on cognitive function, particularly in individuals with Mild Cognitive Impairment (MCI) (Table 2).

The prospective cohort study by Karbownik et al. examined the association between fermented food consumption and cognitive function in 372 medical students under psychological stress. High intake of fermented foods was associated with more severe depressive and anxiety symptoms, highlighting the complex relationship between diet, mental health, and cognitive performance [72].

Another study investigated the effects of fermented Korean ginseng extract-containing drink on stress, fatigue, and sleep in adult workers. Although the overall results did not show significant differences between groups, subgroup analysis revealed improvements in mental fatigue among middle-aged and older subjects, suggesting potential age-related effects [68].

An experimental study on tempeh consumption and cognitive improvement in individuals with mild cognitive impairment (MCI) provided insights into the cognitive benefits of fermented foods. Both tempeh A and tempeh B consumption demonstrated improvements in global cognitive function, with tempeh A showing additional benefits in the language domain [73].

## Future Directions in Research

To date, there is very limited knowledge about how different fermented vegetables affect the gut microbiome, and further, the specific effects of these changes on host health. This information is vital for developing tailored fermented vegetable products. It is possible that in the future, specific strains of fermented vegetables, or pre- and probiotic vegetable products could be developed to achieve certain changes in gut microbiota in order to promote a good health outcome [76]. Although this is not currently achievable, advancing technologies in both fermented food production and gut microbiome analysis mean that it will be possible in the future.

### Strain-Specific Probiotics for Enhanced Benefits

The potential for the health benefits of probiotics depends on the selection of the most appropriate probiotic strains. With the identification of the health-promoting attributes of specific strains, it is now possible to select strains for various health conditions. This much more measured and exact approach to probiotic selection and use is a major shift from the current situation, where many commercially available probiotics have undefined taxonomic status and unproven health benefits [77]. Unfortunately, few if any of the fermented vegetables available on the market today have specific probiotic strains added, or these have not been adequately defined. Typically, the probiotics used are lactic acid bacteria with the general perception that they are safe and promote health. However, without specific identification and testing of strains, we cannot be sure of the probiotic’s safety or health-promoting properties [78]. This is particularly relevant for fermented vegetables as many of their health benefits to date have been attributed to the preservative effect of organic acids and have not been comprehensively linked to the actions of the probiotics present. Probiotics are thought to have health benefits through their effects on the host and/or pathogenic microorganisms, the modulation of gut microbiota, and fermentation of substrates to produce metabolites. The specific strain of a probiotic will influence what proteins, glycans, and organic acids it produces, the specificity and efficacy of interactions with host and pathogenic microbes. Therefore, it is possible that vegetables with a specific health benefit or attribute may be fermented with a strain of the probiotic, matching the criteria for the desired outcome. It is also possible that the addition of a specific probiotic strain to a fermented vegetable that has health benefits attributed to it will enhance the effect by virtue of the interactions between two different substrates of organic acids and bacterially derived enzymes [79]. This is currently speculation, but studies using fermented vegetables with particular probiotics and assessing the effect on host and microbial substrates are most achievable with the availability of modern molecular techniques. This has potential for the development of vegetables that are probiotic carriers with the combination enhancing the effect of both to promote health.

### Exploring the Effects of Fermented Vegetables on Different Populations

Research investigating how fermented vegetables affect different populations is of critical importance to public health. Understanding the effect of fermented foods on different races/ethnicities, both genders, age groups, and those with immune deficiencies is necessary to implement dietary recommendations and therapies involving fermented foods that are specific for different groups [80]. Understanding how specific fermented foods and their components affect these individuals is important to assess whether these foods can be used as a form of therapy for such conditions. A recent review highlighted the paucity of data specific to the effects of fermented foods on those with autoimmune and immune-inflammatory conditions, suggesting that there is still much research that needs to be undertaken in this area [81].

## Conclusions

Psychobiotics derived from fermented vegetables offer a promising avenue for understanding and improving mental health through the gut microbiome. This review has underscored the intricate interplay between gut bacteria and psychological well-being, highlighting how probiotic-rich fermented vegetables can influence mood, cognition, and stress response via the gut-brain axis. From their role in neurotransmitter production to their modulation of immune function and inflammation, psychobiotics offer multifaceted mechanisms through which they may promote mental wellness. Clinical studies have shown encouraging results, indicating that consumption of fermented vegetables can improve mood, alleviate anxiety, and enhance cognitive function in both healthy individuals and those with psychiatric conditions. However, challenges such as the standardization of probiotic strains and optimal dosing regimens remain. Addressing these challenges will be pivotal in advancing psychobiotics from a promising concept to practical therapeutic strategies that benefit individuals’ mental health. Looking ahead, future research should focus on exploring strain-specific probiotics and conducting large-scale clinical trials. By deepening our understanding of these mechanisms and refining interventions, we can harness the potential of fermented vegetables and their psychobiotic properties to foster mental resilience and well-being in diverse populations.

## Data Availability

No datasets were generated or analysed during the current study.
